# Is dragging a drag or is trapping a trap? A comparison of two methods for collecting *Amblyomma americanum* ticks in sites near the species range boundary

**DOI:** 10.1007/s10493-024-00977-6

**Published:** 2024-12-09

**Authors:** Peter Briggs, Lawson Trimmell, Javier D. Monzón

**Affiliations:** Natural Science Division, Pepperdine University, 24255 Pacific Coast Highway, Malibu, CA USA

**Keywords:** Lone star tick, *Amblyomma americanum*, Dragging, CO_2_ trapping, Range expansion

## Abstract

The incidence of tick-borne diseases in the United States has more than doubled since the early 2000s. Research on ticks is a priority to mitigate the spread of tick-borne diseases. Thus, it is important to understand how to efficiently collect large numbers of ticks for studies of genetics, behavior, physiology, vector competence, tick repellants, and acaricides. In this study, we compared the efficiency of two methods—dragging and CO_2_-baited trapping—for collecting lone star ticks (*Amblyomma americanum*) across two distinct regions of its expanding range. We performed simultaneous dragging and trapping collections at six sites, three in Oklahoma and three in New York and New Jersey, USA. Our results demonstrate that dragging was more efficient than trapping for collecting lone star tick nymphs. However, dragging and trapping were similar in efficiency for collecting adult males and females. There were no regional differences in trapping or dragging efficiency. Additionally, we discuss material and labor costs of each method to inform researchers who need to rapidly collect as many ticks as possible with the most efficient and cost-effective method.

## Introduction

**Fig. 1:**
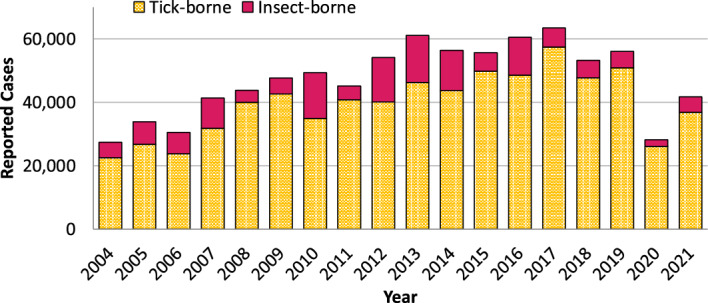
Reported cases of vector-borne human diseases in the United States (2004–2021). Reportable tick-borne diseases include Lyme disease, anaplasmosis/ehrlichiosis, spotted fever rickettsiosis, babesiosis, tularemia, and Powassan virus. Reportable insect-borne diseases include Dengue viruses, Zika virus, West Nile virus, Malaria, Chikungunya virus, California serogroup viruses, encephalitis viruses, yellow fever virus, and plague. Data: Centers for Disease Control and Prevention

Ticks transmit disease pathogens more frequently than other arthropod vectors in North America and Europe. From 2004 to 2021, over 710,000 cases of tick-borne diseases were reported in the United States, greatly outnumbering insect-borne diseases in this period (CDC 2021). The incidence of tick-borne diseases in the United States has more than doubled since the early 2000s, although there was a notable decrease in 2020 (Fig. 1). The substantial decrease in reported tick-borne diseases in 2020 likely reflects an outcome of the COVID-19 pandemic instead of a true decrease in disease incidence; this is possibly due to pandemic-related stay-at-home orders that reduced outdoor activities, hesitation to seek healthcare following a tick bite and the onset of symptoms, or changes in the way that febrile illnesses were diagnosed and treated (McCormick et al. 2021; Arahirwa et al. 2023). Surveillance and research on ticks are crucial to understand and mitigate the spread of tick-borne pathogens. Many researchers conduct their studies on ticks that have been raised in a laboratory for many generations (e.g., Wright et al. 2015; Portugal III and Goddard 2016; Dubie and Noden 2023; Nielebeck et al. 2023; Rochlin et al. 2024). However, lab-raised tick populations can be genetically different from natural populations of the same species (Monzón et al. 2016). Additionally, ongoing research in our lab is exploring behavioral and physiological differences between lab-raised ticks and wild-caught ticks from distinct geographic populations (Kim et al. 2022; Nielebeck et al. 2022, 2023). These biological differences between wild-caught ticks and lab-raised ticks of the same species emphasize the importance of using wild-caught ticks in studies of genetics, behavior, physiology, vector competence, tick repellants, and acaricides.

The two most common methods for collecting ticks from the environment are dragging and CO_2_ trapping (Petry et al. 2010; Mays et al. 2016). Dragging involves a researcher pulling a white fabric over grass or leaf litter and routinely collecting questing ticks that attach to the fabric (Solberg et al. 1992; Estrada-Peña et al. 2013). Trapping takes advantage of ticks’ natural attraction to CO_2_ and uses sublimating dry ice to lure ticks to a canvas where they get stuck on tape (Wilson et al. 1972). Although dragging is an effective method for collecting some tick species belonging to various genera, CO_2_ trapping appears to be particularly effective for collecting ticks belonging to genus *Amblyomma* (Wilson et al. 1972; Guglielmone et al. 1985; Norval et al. 1987; Barré et al. 1997; Schulze et al. 1997; de Paula et al. 2022).

The lone star tick (*Amblyomma americanum*) is an aggressive species of hard tick in family Ixodidae. All active developmental stages bite humans and numerous other mammalian and avian hosts (Bishopp and Trembley 1945). The lone star tick is an arthropod of medical and veterinary importance in North America because it is a vector of several pathogens of humans, domestic animals, and wild animals (Childs and Paddock 2003; Goddard and Varela-Stokes 2009). The lone star tick is a competent vector of pathogenic bacteria such as *Ehrlichia* spp., *Rickettsia* spp., and *Francisella tularensis* (Childs and Paddock 2003; Levin et al. 2017; Rochlin and Toledo 2020); protozoa such as *Theileria* spp. (Almazán et al. 2022); and emerging viruses such as Bourbon and Heartland viruses (Tokarz et al. 2018; Dupuis et al. 2023). Additionally, lone star tick bites are associated with the onset of alpha-gal syndrome (red meat allergy) and southern tick-associated rash illness (STARI) (Commins et al. 2011; Feder et al. 2011). The geographic range of the lone star tick has been expanding northward and westward since the mid-1900s. This range expansion appears to result from widespread ecological changes in North America, such as extension of second-growth forest and increase in white-tailed deer (*Odocoileus virginianus*) populations (Rochlin et al. 2022, 2023).

Although several studies have evaluated the effectiveness of various tick collection methods, these studies mostly focused their evaluations on sampling biases associated with the different methods to better estimate tick densities and inform tick surveillance programs. Our study, on the other hand, aims to uncover which method is most efficient for collecting large numbers of ticks that can be used for research purposes, such as genetic surveys or behavioral experiments that require wild ticks. Additionally, previous studies were conducted at a local scale. Our study is the first to compare dragging and trapping in multiple distinct regions of the expanding range boundaries of the lone star tick, and it is one of the only studies analyzing method efficiency in terms of ticks per hour, a standardization recommended by Estrada-Peña et al (2013). This time-based approach to understanding method efficiency disregards the relevance of standardizing area sampled, allowing researchers to relocate a dragging effort or even a trap, to maximize the number of ticks collected. This is especially important because ticks are usually aggregated and not uniformly distributed throughout a given site (Schulze et al. 2001; Estrada-Peña et al. 2013); hence, understanding the differences in cost and method effectiveness can reduce wasted effort when the goal is to collect large numbers of ticks. This study aims to inform researchers who need to rapidly collect as many lone star ticks as possible with the most efficient and cost-effective method.

## Methods

### Study sites

**Fig. 2:**
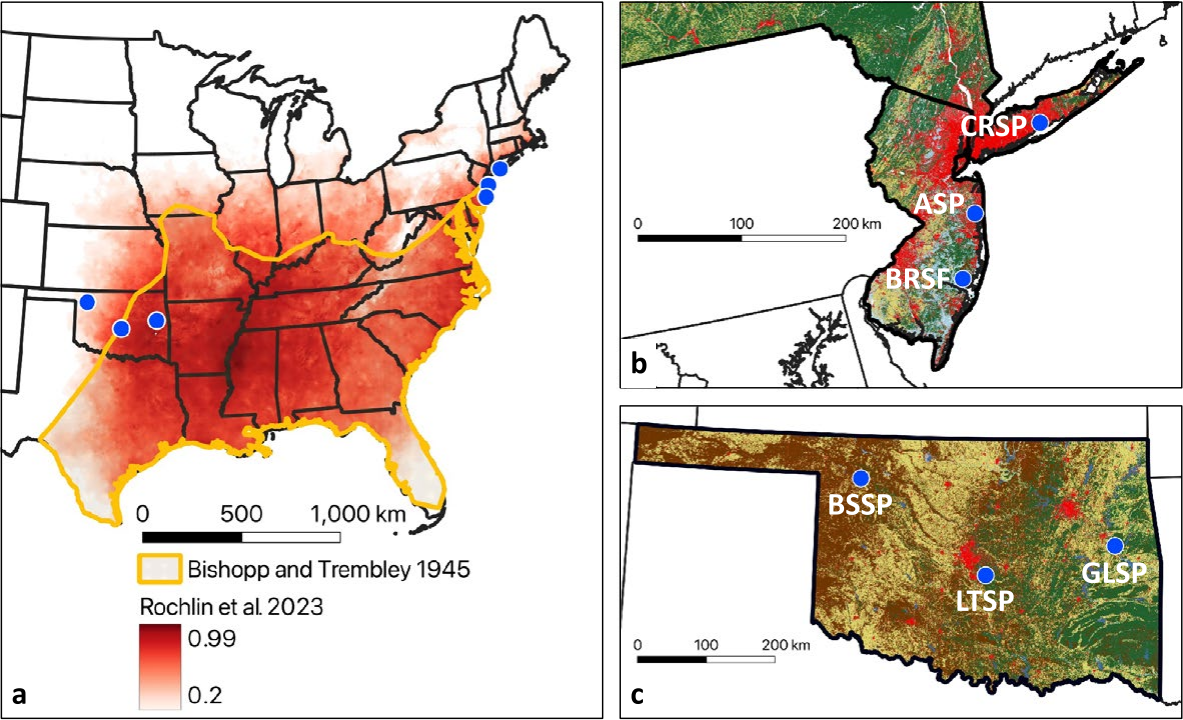
**a** Geographic range of lone star tick in the United States. Yellow = approximate boundaries of historical species distribution, as estimated by Bishopp and Trembley (1945). Red = approximate current species distribution, with shades of red indicating probability of occurrence, from 0.20 to 0.99, as modeled by Rochlin et al. (2023). **b** Northeastern sampling sites in New York and New Jersey. CRSP = Connetquot River State Park; ASP = Allaire State Park; BRSF = Bass River State Forest. **c** Western sampling sites in Oklahoma. BSSP = Boiling Springs State Park; LTSP = Lake Thunderbird State Park; GLSP = Greenleaf State Park. ﻿Land cover maps of New York, New Jersey, and Oklahoma based on satellite imagery from US Geological Survey National Land Cover Database

This study was conducted at six sites in two regions representing distinct expansion fronts of the lone star tick separated by more than 1800 km (Fig. 2a). On the northeastern expansion front, three sites were spread south to north across New Jersey (NJ) and New York (NY): Bass River State Forest in Burlington County, Allaire State Park in Monmouth County, and Connetquot River State Park in Suffolk County (Fig. 2b). On the western expansion front, three sites were spread east to west across Oklahoma (OK): Greenleaf State Park in Muskogee County, Lake Thunderbird State Park in Cleveland County, and Boiling Springs State Park in Woodward County (Fig. 2c). Connetquot River State Park in Long Island, NY and Boiling Springs State Park in western OK lie outside the historical range boundary where the species was known to occur in the mid-1900s (Bishopp and Trembley 1945).

In western OK, Boiling Springs State Park is a pocket of evergreen forest surrounded by relatively arid grassland; we sampled in forested and open grassy areas. Lake Thunderbird State Park is composed of deciduous forest with patches of grassland located in central OK; we sampled primarily in grassy corridors and forest ecotones near the lakefront. Greenleaf State Park features a combination of deciduous and mixed forest in eastern OK; we focused our collection efforts on grassy powerline corridors surrounded by forest. In NY, Connetquot River State Park is primarily deciduous forest with thick brush understory; we sampled grassy corridors directly bordering the forest near a residential neighborhood. In central NJ, Allaire State Park is a combination of mixed and deciduous forests; we sampled the forest floor and the boundaries of a large grassy field. Bass River State Forest, in southern NJ, is characterized by pine barrens; we sampled amidst a combination of pitch pine-dominated forest and mixed oak-pine forest adjacent to Lake Absegami.

### Field collection and species identification

All field sampling occurred in late May 2023 on days that were sunny and without recent rainfall. At each site, all dragging and trapping efforts occurred concurrently during midday between approximately 11:00 and 13:00 h, except for Greenleaf State Park, in which collections occurred between approximately 13:00 and 15:00 h. The ambient temperature of all days was between 17 °C and 24 °C during field collections. We performed three trapping events and three dragging events simultaneously at every site for a total of 36 collection events over the span of the study. Traps were constructed with a similar design as described by Wilson et al. (1972). Specifically, each trap was baited with approximately 1.4 kg of dry ice contained in a Styrofoam cooler (26×21×17 cm) with a lid and one 3-cm hole on each side of the cooler for CO_2_ to diffuse outwards and sideways across the forest floor. The cooler sat on a canvas of approximately one square meter with double-sided tape surrounding the cooler to trap attracted ticks. Duration of trapping events ranged from 115 to 168 min and dry ice remained at the end of the trapping period. Each drag was constructed by BioQuip Products Inc. and consisted of a white 100% cotton sailcloth canvas of approximately one square meter fastened to a wooden dowel with a long rope attached to each end. We used this rope to pull the cloth across grass and leaf litter, stopping every 5 to 10 m to examine both sides of the cloth for ticks while periodically examining and sampling from our own white tick suits (theticksuit.com). Duration of dragging events ranged from 80 to 130 min. We collected adult ticks alive in snap-cap tubes and nymphs in ethanol.

In the laboratory, we sorted all collected ticks by site, species, life stage, and sex under dissection microscopes. We used pictorial keys (Dubie et al. 2017; Egizi et al. 2019) to identify adults to species and sex and to identify nymphs to species.

### Statistical analysis

We calculated the collection rate in ticks per hour by dividing the number of ticks collected by either the number of hours spent continuously dragging or by the number of hours between when a trap was set and when it was removed. This rate provided a meaningful and comparable metric between collection methods. We performed Wilcoxon signed-rank tests with Holm-Bonferroni adjustment for multiple comparisons to analyze differences between collection methods for each group of ticks in each region. We plotted the data and performed all statistical tests is R (R Core Team 2023) using packages tidyr (Wickham et al. 2024), tidyverse (Wickham et al. 2019), ggplot2 (Wickham 2016), ggimage (Yu 2023), and gridExtra (Auguie 2017).

## Results

### Collection demographics

In 34 drag-hours and 41 trap-hours, we collected a total of 1,954 ticks of four species (Table 1). The four species, in descending order of collection size, were lone star tick (*A. americanum*) (n = 1769), blacklegged tick (*Ixodes scapularis*) (n = 165), Asian longhorned tick (*Haemaphysalis longicornis*) (n = 11), and American dog tick (*Dermacentor variabilis*) (n = 9). Most collected ticks were nymphs (n = 1257), while adult males (n = 316) and adult females (n = 381) had relatively similar representation. *Amblyomma americanum* adults and nymphs were collected in all six sites by both dragging and trapping. *Ixodes scapularis* adults and nymphs were collected only in northeastern sites, but primarily by dragging. *Dermacentor variabilis* adults were collected in two western and two northeastern sites, also primarily by dragging. *Haemaphysalis longicornis* nymphs were collected only in Allaire State Park and only by dragging; this collection represents an early report in Monmouth County, NJ, where the invasive *H. longicornis* tick species established recently.

**Table 1:** Numbers of ticks collected by dragging and trapping at three sites in Oklahoma and three sites in New York and New Jersey

Site	Method	n	Time (min)	*A. americanum*		*I. scapularis*		*D. variabilis*		*H. longicornis*	
				Adults	Nymphs	Adults	Nymphs	Adults	Nymphs	Adults	Nymphs
Greenleaf State Park, OK	Drag	3	240	29	45	0	0	1	0	0	0
	Trap	3	380	75	42	0	0	0	0	0	0
Lake Thunderbird State Park, OK	Drag	3	350	96	271	0	0	0	0	0	0
	Trap	3	351	48	172	0	0	0	0	0	0
Boiling Springs State Park, OK	Drag	3	360	18	71	0	0	1	0	0	0
	Trap	3	465	135	30	0	0	1	0	0	0
Connetquot River State Park, NY	Drag	3	345	41	68	0	50	1	0	0	0
	Trap	3	390	62	48	0	4	0	0	0	0
Allaire State Park, NJ	Drag	3	371	51	134	2	66	5	0	0	11
	Trap	3	415	28	34	0	6	0	0	0	0
Bass River State Forest, NJ	Drag	3	390	35	119	1	31	0	0	0	0
	Trap	3	456	67	50	0	5	0	0	0	0
	Total	36	4513	685	1084	3	162	9	0	0	11

n = number of independent dragging or trapping events at each site

### Comparison of collection methods

**Fig. 3:**
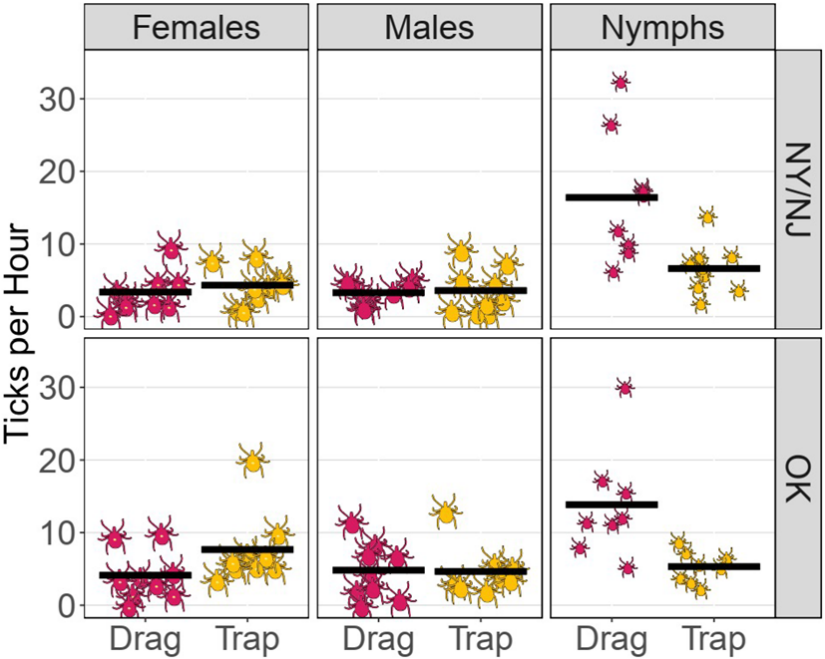
Comparison of efficiency of dragging and CO_2_ trapping for collecting adult female, adult male, and nymphal lone star ticks in New York/New Jersey and Oklahoma. Each tick icon represents one collection event. Horizontal bars represent group means. After applying a Holm-Bonferroni adjustment for multiple comparisons, there were no statistically significant differences between methods in collecting efficiency for adult lone star ticks (p > 0.03), but dragging was more efficient for collecting nymphs in New York/New Jersey (p = 0.003) and in Oklahoma (p = 0.014)

Since lone star ticks of nymph and adult life stages comprised more than 90% of ticks collected, we focused the comparison of collection methods only on the lone star tick (Fig. 3). After applying a Holm-Bonferroni adjustment for multiple comparisons, there were no statistically significant differences between methods in collecting efficiency for adult lone star ticks (NY/NJ females: p = 0.489; NY/NJ males: p = 0.863; OK females: p = 0.038; OK males: p = 0.931). The modestly higher average of adult OK females collected by trapping was likely an artifact of a single trap that collected an unusually high number of females in Boiling Springs State Park. In contrast, nymphs were more consistently and efficiently collected by dragging compared to trapping. In NY and NJ, dragging yielded an average of 16.4 nymphs/hour, while trapping yielded an average of 6.6 nymphs/hour (p = 0.003). Likewise, in OK, dragging yielded an average of 23.3 nymphs/hour, while trapping yielded an average of 13.5 nymphs/hour (p = 0.014). These higher averages in OK were elevated by a single drag sample that collected 99 nymphs/hour and a single trap sample that collected 79 nymphs/hour, both at Lake Thunderbird State Park, OK. Excluding these outliers, dragging in OK yielded an average of 13.8 nymphs/hour while trapping yielded an average of 5.3 nymphs/hour (p = 0.003). Thus, for collecting lone star tick nymphs, dragging was approximately 2.5 times more efficient than trapping in NY/NJ and approximately 1.7 times more efficient than trapping in OK. There were no significant differences in trapping efficiency or dragging efficiency by region (p > 0.05).

## Discussion

The goal of this study was to compare the efficiency of dragging and trapping with dry ice for collecting lone star ticks from two regions of geographic expansion. Our results demonstrate that dragging is more efficient than trapping for collecting lone star tick nymphs. However, dragging and trapping are similar in efficiency for collecting adult males and females. These patterns are consistent across geographic regions.

Other studies have compared various methods of collecting lone star ticks in the field, including dragging, CO_2_ trapping, flagging, sweep netting, walking, and collecting from hosts (Table 2). Even Wilson et al. (1972), who first introduced the method of attracting lone star ticks to a trap baited with sublimating dry ice, compared their novel technique with flagging, which was already a conventional technique at the time. They concluded that trapping produced a more accurate estimate of tick abundance in an area than flagging. This is because flagging is dependent on the physical environment of the area sampled, whereas CO_2_ trapping is more consistent regardless of the physical environment (Wilson et al. 1972). Our results on nymphs are most consistent with those of Schulze et al. (1997), who found that dragging produced more nymphs than CO_2_ trapping; however, they also found that CO_2_ trapping produced more adults than dragging, a pattern we did not observe. Solberg et al. (1992) observed that CO_2_ trapping was superior to dragging for collecting every host-seeking life stage of lone star tick, but they set out their traps for 24 h. Similarly, Mays et al. (2016) compared six different methods and found that CO_2_ trapping was the superior method for collecting lone star ticks, but they also operated dry ice traps overnight. Interestingly, Petry et al. (2010) collected more adults and nymphs by trapping compared to dragging, even though they only set their dry ice traps for 1 h. Rynkiewicz and Clay (2014) observed the opposite: they collected more adults and nymphs by dragging compared to trapping, even though they re-baited their traps with dry ice each morning and evening. Our study is the first to compare tick collection methods in two geographic regions separated by more than 1800 km and sampling sites within a region separated by an average of 190 km. As such, we were limited to one afternoon per sampling site and our goal was to obtain as many ticks as possible. Our results are thus most relevant to researchers with similar limitations and similar goals.

**Table 2:** Studies comparing various methods of collecting lone star tick, *Amblyomma americanum*. Trapping refers to CO_2_-baited trapping with contained dry ice

Reference	Location	Methods Studied	Results
Wilson et al. (1972)	Oklahoma (eastern)	Trapping Flagging	Trapping required less manpower than flagging, particularly in areas with high tick abundance Trapping produced a more accurate estimate of tick abundance in an area than flagging Trapping attracted adults from up to 21.3 m away
Koch and McNew (1981)	Oklahoma (eastern)	Uncontained dry ice Dry ice in foam bucket Dry ice in wooden box Dry chemical trap Live rabbit trap	Dry ice (uncontained or contained) was the most effective Dry chemical trap was nearly as effective as dry ice trap Live rabbit trap was ineffective
Ginsberg and Ewing, (1989)	New York (southern)	Trapping Flagging Walking Collecting from hosts	Overnight trapping collected more total nymphs than flagging, but flagging collected more nymphs/hour Trapping was particularly effective for *A. americanum* ticks due to their rapid mobility Flagging and walking collected adults in late spring and early summer No active life stages were collected from rodent hosts (*Peromyscus*)
Kinzer et al (1990)	Oklahoma (eastern)	Trapping Flagging	Trapping collected significantly more adults than flagging, regardless of habitat and month Trapping collected significantly more nymphs than flagging, but habitat and month significantly affected capture of nymphs Trapping collected significantly more larvae than flagging, but methods were not significantly different during late spring and early summer Trapping is more precise than flagging, but advantage of trapping is more pronounced during late-season sampling
Solberg et al. (1992)	New Jersey (central)	Dragging Trapping Walking	Trapping was superior to dragging and walking for every host-seeking life stage Trapping attracted adults from up to 5 m away
Schultze et al. (1997)	New Jersey (central)	Dragging Trapping Walking Pitfall traps Collecting from leaf litter	CO_2_ trapping produced more adults than dragging, but dragging produced more nymphs and larvae than CO_2_ trapping Dragging produced more adults, nymphs, and larvae than walking Pitfall traps and leaf litter samples collected very few ticks
Petry et al. (2010)	Missouri (northern)	Dragging Trapping	Trapping was more effective than dragging for capturing adults, particularly in forest habitat Trapping was more effective than dragging for capturing nymphs, regardless of habitat type Dragging captured more larvae than trapping due to slow response time and reduced mobility of larvae
Rynkiewicz and Clay, (2014)	Indiana (southern)	Dragging Trapping Collecting from hosts	Dragging was more effective than trapping for both adults and nymphs, but this difference was more pronounced in open habitat compared to forest habitat No active life stages were collected from rodent hosts (*Peromyscus* and *Microtus*)
Mays et al. (2016)	Tennessee (western)	Dragging Trapping Flagging CO_2_ dragging CO_2_ flagging Sweep netting	CO_2_ dragging and CO_2_ flagging were comparable to traditional dragging and flagging Trapping was the most consistently superior method, especially in ﻿upland deciduous and coniferous forests Sweep netting was significantly less effective than the other methods
Espada et al. (2021)	Virginia (eastern)	Dragging denim Dragging corduroy Flagging denim Flagging corduroy	Flagging was more effective than dragging for collecting adult ticks, but differences between methods were not significant for nymphs Corduroy was more effective than denim for collecting nymphs, but differences between materials were not significant for adults
This study	Oklahoma, New York, New Jersey	Dragging Trapping	Dragging was more efficient than trapping for nymphs Dragging and trapping were equally efficient for adult males and females There were no regional differences in trapping efficiency or dragging efficiency

Results summarized here are only pertinent to lone star tick

Dry ice traps are effective for sampling lone star tick because this species is an aggressive pursuer of hosts. The lone star tick has a keener proclivity to quest, not just vertically up and down the vegetation, but also horizontally as it follows CO_2_ and thermal gradients in pursuit of potential hosts (Wilson et al. 1972; Otálora-Luna et al. 2022). For example, lone star tick nymphs consistently won races against blacklegged tick nymphs in the laboratory (Ginsberg and Ewing 1989). The increased horizontal mobility of lone star tick compared to other species accounts for the bias of sampling lone star tick using dry ice traps (Ginsberg and Ewing 1989; Schulze et al. 1997; Petry et al. 2010). Similarly, the increased mobility of lone star tick adults compared to lone star tick nymphs accounts for the bias of sampling adults using dry ice traps (Kensinger and Allan 2011). Thus, dry ice trapping can effectively attract adults from a distance of 5 m in 24 h (Kensinger and Allan 2011) and even up to 21 m if given enough time (Wilson et al. 1972). However, if trapping is limited to shorter durations, as was the case in our study, trapping is no more efficient in capturing adults than dragging. Conversely, dragging was more efficient for capturing nymphs because of their limited mobility. For the same reason, dragging is the most effective method for capturing larvae (Petry et al. 2010). Our study does not address weather-related limitations of each collecting method. How precipitation or wind impact trapping versus dragging is an important issue that merits further investigation.

Dragging and trapping can further be compared based on costs associated with materials and labor. The only material cost for dragging is the cost of the drags themselves, which are relatively inexpensive—about $25 to $45 USD per drag if purchased from an entomology equipment supplier, or less if constructed from the materials purchased separately. The material costs for trapping include the costs of the canvas ground cover, cooler, double-sided tape, and dry ice. Dry ice is particularly challenging because it represents a recurring cost and must be purchased close to the time of field sampling. We used approximately 1.4 kg of dry ice per trap, which cost approximately $13 USD per trapping event. The recurring cost of dry ice makes trapping the more expensive collection method in material costs, and the difference becomes more pronounced when placing many traps. Additionally, dry ice suppliers are limited, especially in remote areas. As for labor costs, it takes about 5 min to set up a trap, and 5–20 min to collect ticks from the trap at the end of the trapping period. CO_2_-baited traps can thus be armed and attract ticks for potentially several hours, as long as there is dry ice to sublimate. The trap works independently of researcher involvement between set-up and take-down periods. Dragging, on the other hand, requires constant active labor in the form of a researcher walking and examining the drags. The researcher must be present and participating for the entire duration of the dragging event. Whereas one researcher can set up many traps simultaneously to sample ticks from a large area, several researchers are required to drag simultaneously to sample ticks from a large area. The recurring cost of manpower makes dragging the more expensive collection method in labor costs, and the difference becomes more pronounced when performing many drags.

Overall, dragging is the most efficient method if the goal is to collect lone star tick nymphs or questing ticks of multiple species. But if the goal is to collect adult lone star ticks, we recommend combining CO_2_ trapping and dragging because they are equally efficient, so setting CO_2_ traps while dragging can effectively double one’s collecting effort in the field.

## Data Availability

The datasets generated and analysed during the current study are available from the corresponding author upon request.
